# Markov State Modelling of Disease Courses and Mortality Risks of Patients with Community-Acquired Pneumonia

**DOI:** 10.3390/jcm9020393

**Published:** 2020-02-05

**Authors:** Jens Przybilla, Peter Ahnert, Holger Bogatsch, Frank Bloos, Frank M. Brunkhorst, SepNet Critical Care Trials Group, PROGRESS study group, Michael Bauer, Markus Loeffler, Martin Witzenrath, Norbert Suttorp, Markus Scholz

**Affiliations:** 1 Institute for Medical Informatics, Statistics and Epidemiology (IMISE), Universität Leipzig, Härtelstr. 16-18, 04107 Leipzig, Germany; peter.ahnert@imise.uni-leipzig.de (P.A.); holger.bogatsch@imise.uni-leipzig.de (H.B.); markus.loeffler@imise.uni-leipzig.de (M.L.); markus.scholz@imise.uni-leipzig.de (M.S.); 2 German Center for Lung Research (DZL), Aulweg 130, 35392 Gießen, Germany; martin.witzenrath@charite.de (M.W.); norbert.suttorp@charite.de (N.S.); 3 Clinical Trial Centre Leipzig, Universität Leipzig, Härtelstr. 16-18, 04107 Leipzig, Germany; 4 Department of Anesthesiology and Intensive Care Medicine, Jena University Hospital, Am Klinikum 1, 07747 Jena, Germany; frank.bloos@med.uni-jena.de (F.B.); frank.brunkhorst@med.uni-jena.de (F.M.B.); michael.bauer@med.uni-jena.de (M.B.); 5 Center for Sepsis Control & Care (CSCC), Jena University Hospital, Am Klinikum 1, 07747 Jena, Germany; 6 Center for Clinical Studies, Jena University Hospital, Salvador-Allende-Platz 27, 07747 Jena, Germany; 7 SepNet Critical Care Trials Group c/o Sepsis-Stiftung, Carl-Zeiß-Str. 12, 07743 Jena, Germany; info@sepsis-stiftung.de; 8 Department of Infectious Diseases and Respiratory Medicine, Charité—Universitätsmedizin Berlin, corporate member of Freie Universität Berlin, Humboldt-Universität zu Berlin, Charitéplatz 1, 10117 Berlin, Germany; progress-study-group@imise.uni-leipzig.de; 9 Division of Pulmonary Inflammation, Charité—Universitätsmedizin Berlin, corporate member of Freie Universität Berlin, Humboldt-Universität zu Berlin, and Berlin Institute of Health, Charitéplatz 1, 10117 Berlin, Germany

**Keywords:** community-acquired pneumonia, prognosis, sepsis, SOFA score, stochastic model, continuous-time Markov model, medical decision making

## Abstract

Community-acquired pneumonia (CAP) is one of the most frequent infectious diseases worldwide, with high lethality. Risk evaluation is well established at hospital admission, and re-evaluation is advised for patients at higher risk. However, severe disease courses may develop from all levels of severity. We propose a stochastic continuous-time Markov model describing daily development of time courses of CAP severity. Disease states were defined based on the Sequential Organ Failure Assessment (SOFA) score. Model calibration was based on longitudinal data from 2838 patients with a primary diagnosis of CAP from four clinical studies (PROGRESS, MAXSEP, SISPCT, VISEP). We categorized CAP severity into five disease states and estimated transition probabilities for CAP progression between these states and corresponding sojourn times. Good agreement between model predictions and clinical data was observed. Time courses of mortality were correctly predicted for up to 28 days, including validation with patient data not used for model calibration. We conclude that CAP disease course follows a Markov process, suggesting the necessity of daily monitoring and re-evaluation of patient’s risk. Our model can be used for regular updates of risk assessments of patients and could improve the design of clinical trials by estimating transition rates for different risk groups.

## 1. Introduction

Community-acquired pneumonia (CAP) is the most frequent cause of death among infectious diseases worldwide and a very frequent cause of hospital admissions in developed countries, with 289,633 hospitalizations for CAP in 2018 in Germany [1]. Lethality of hospitalized CAP has remained high at around 13% in developed countries [2,3]. The course of CAP in the hospital can be heterogeneous and highly dynamic with disease deteriorations frequently occurring within a few hours and requiring immediate intensive care [4]. In the context of CAP, several scoring systems have been proposed. For evaluation of CAP severity in regard to mortality, CURB-65 [5,6] and PSI [7] have been well established. SCAP [8] has been developed to support clinical prediction of severe CAP. SMART-COP [9] and IDSA/ATS minor criteria [10] predict need for intensive care treatment, while criteria according to Halm [11,12] allow evaluation of reaching clinical stability. CURB-65 and IDSA/ATS major and minor criteria are part of the German CAP guidelines [13]. In patients with sepsis, monitoring of IDSA/ATS minor criteria and organ function has been suggested [2,3,14,15]. In contrast, a general scheme of daily risk reevaluation for CAP patients is not established. As mortality risk increases from 6% for improving CAP to 34% for nonresolving CAP or clinical failure [16], regular updates of patient’s risk evaluation during a hospital stay are warranted. 

We here propose a stochastic mathematical model of the disease course of hospitalized CAP patients of different initial disease severity including prediction of mortality. In particular, we choose continuous-time Markov modelling, which was used in different disease contexts. One of the first papers proposing this type of model for medical applications was Chiang [17]. Since then, Markov models proved to be a valuable tool in medicine to describe and analyze time courses of different diseases such as cancer survival [18], survival after heart transplantation [19], or more recently, success of incontinence treatment procedures [20] and repeated hospitalization and death in heart failure patients [21]. In the field of infectious diseases, the concept was applied to modelling of immunologic states of HIV patients [22] and sepsis severity [23]. The major idea of this modelling approach is to describe random transitions between different disease states and to calculate corresponding sojourn times. Given a sufficiently rich database for testing model assumptions, for parametrizing the model, and for validation, a quantitative model of disease progression can be established which allows updating patient’s risk evaluation at any time. 

We here consider disease states based on the sequential organ failure assessment (SOFA) score, which recently was shown to be a good operationalization of CAP severity [24]. We used time series data of CAP patients with different initial disease severity taken from four large clinical trials to calibrate our model and to validate its predictions. 

## 2. Materials and Method

### 2.1. Patient Data

We used time series data of CAP patients taken from the clinical observational trial PROGRESS [25] and three randomized controlled clinical trials of severe sepsis patients (MAXSEP [26], VISEP [27], SISPCT [28]). 

PROGRESS is an ongoing observational study with the purpose of identifying predictors for the severity of the disease course in CAP patients. Observation time was day of enrolment and four consecutive days. Follow-up information on survival was available for nearly all patients after 28 days. By initial study design, a subcohort of 142 severely ill CAP patients (ssCAP cohort) was documented only on the day of enrolment and at follow up. Data of these patients could not be used for model development but were used for model validation regarding prediction of 28 d mortality. 

Of three SepNet studies conducted to analyze different treatment strategies for severe sepsis or septic shock, patients with sepsis due to CAP (MAXSEP, SISPCT) or community-acquired respiratory tract infections (VISEP) were considered for the present analysis. In all three studies, patients were monitored daily until death, discharge, transfer to another hospital, or the end of the maximum observation time of 22 days. Mortality was assessed after 28 days and after 90 days. None of the SepNet studies showed significant differences between study arms regarding their respective primary endpoints (28 days mortality or mean SOFA). Therefore, we did not distinguish between study arms when analysing the single studies. In Table 1, we provide characteristics of these studies. For more details and metadata we refer to the Leipzig Health Atlas (www.health-atlas.de) and the original publications of these studies.

In our analysis, we use the SOFA score [29] as operationalization of CAP severity [24]. The SOFA score summarizes six subscores evaluating the function of the pulmonary system (oxygenation), the central nervous system (Glasgow Coma Scale), the cardiovascular system (mean arterial pressure or requirement of stimulating treatment), coagulation (platelet counts), liver (bilirubin), and kidney (creatinine). Every subscore is evaluated on a discrete scale of 0 (best) to 4 (worst). Thus, the SOFA score takes values between 0 and 24. Missing values of subscores were imputed applying last observation carried forward (LOCF) method. For the SepNet data, we started with day two after enrolment, because some SOFA subscores were not available at enrolment. 

**Figure A1:**
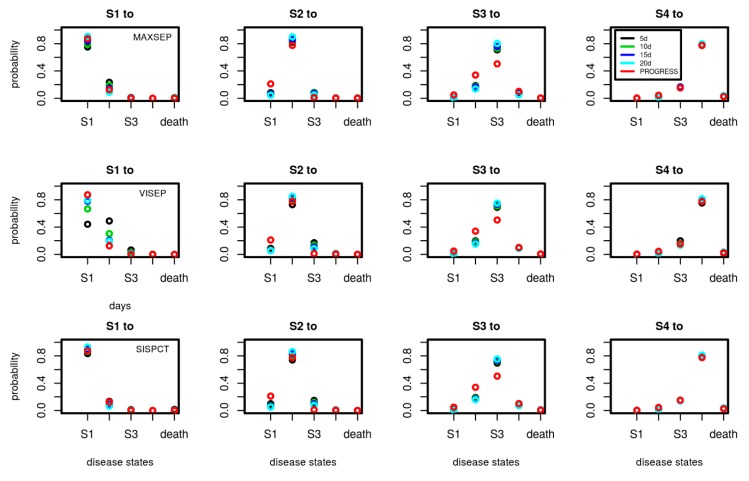
Comparison of transition probabilities between disease states estimated for the SepNet trials in dependence on the number of data points used for calibration (5, 10, 15, and 20 days). Each subfigure shows the daily transition probabilities from one disease state (title of figure) to the other possible disease states shown on the x-axis. Transition probabilities corresponding to calibration on 15 days are also shown in Figure 5 of the main document. We added the transition probabilities of PROGRESS for comparison. Good agreements of transition probabilities between studies and calibration scenarios were observed. An exception is VISEP showing stronger dependence of estimates on the number of time points used for calibration.

For PROGRESS, we used data of all five observation days for calibrating the Markov model. For SepNet studies, sample sizes of pneumonia patients are considerably smaller. Therefore, we experimented with different time intervals (5, 10, 15, and 20 days) for the calibration of the model and decided that 15 days is sufficient (see also Appendix A.2, Figure A1). 

### 2.2. Defining States of CAP Severity

**Figure 1:**
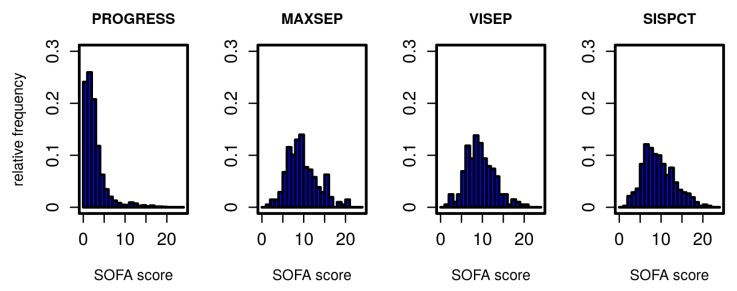
Distribution of SOFA scores for all studies on the first study day.

The Markov model to be established describes transitions between different states of the disease. We define these states based on the SOFA score. In Figure 1, we present the distribution of initial SOFA scores per study. According to inclusion criteria, the distributions of the SOFA scores from SepNet studies were similar, whereas PROGRESS patients showed a considerably less severe initial disease state. To allow estimation of transition probabilities between disease states, it was necessary to form categories based on the SOFA score with sufficiently large allocation numbers. We chose five states corresponding to four SOFA score intervals and death as a final state. SOFA score intervals were chosen in such a way that SOFA distributions of PROGRESS and SepNet data were well represented in each defined disease state (Table 2).

Using this definition, we determined the transition events observed in the first five days in PROGRESS (Table 3) and the first 15 days in the SepNet studies (Table 4). The majority of patients in PROGRESS stay in disease states S1 and S2 or switch between them, whereas in the SepNet studies, the majority of patients are in states S2–S4 with few reaching S1. 

### 2.3. Establishing the Markov Model

**Figure 2:**
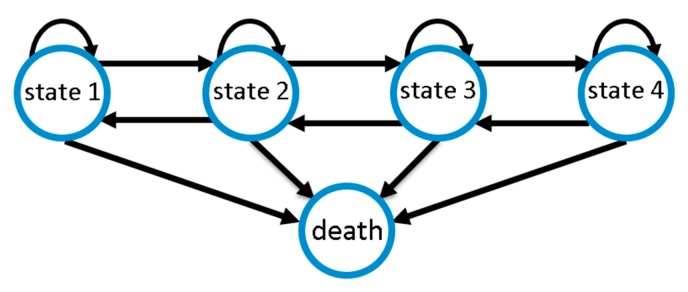
Markov model of community-acquired pneumonia (CAP) disease course with four disease states of increasing severity and death as absorbing state. Disease ameliorations and deteriorations occur stepwise, while death is possible at any state.

We modelled CAP disease course as a Markov state model considering the five disease states defined above. We assume that improvements or deteriorations of disease states occur stepwise; i.e., a deterioration by two disease states requires two deterioration events of one stage each. This is motivated by the fact that changes in SOFA score require the change of certain physiological parameters. By this assumption, the number of free model parameters is significantly reduced, improving identifiability of the remaining parameters. However, since the sojourn times of our disease states are modelled as continuous random variables, deteriorations are allowed to occur rapidly, e.g., overnight, so that changes of more than one disease state within one day are possible. Death serves as an absorbing state of the entire process. Figure 2 illustrates possible transitions. 

The model was parameterized by a matrix of transition intensities Q, which describes the rate of transition events between two states. Via the Chapman Kolmogorov equation, this translates into a matrix of time-dependent transition probabilities between two states. At first, we used data of the first five days of a study to parametrize the model. Since the observation period was considerably longer in SepNet studies, we also performed fittings using data of the first 15 days with improved results. Due to differences between the four studies (see Table 1), we fitted them separately.

To build the model, we used the R package *msm* [30]. Parameters were estimated by maximum likelihood methods, separately for each study. Mathematical details are explained in Appendix A.1. 

### 2.4. Comparisons with Other Risk Scores

By our modelling, a daily re-evaluation of a patient’s risk is supported. We performed analysis of receiver operating characteristics of 28 d mortality to assess the benefit of this approach. For this purpose, we compared the predictive performance of our disease states, CURB-65, and PSI at baseline with those of the model-predicted overall mortality risk during the observed disease courses of patients in PROGRESS. The latter was calculated by multiplying survival probabilities of each observation day, and finally, the survival probability of the transition of day 5 to day 28. Receiver operating characteristic (ROC)analysis and comparison of areas under the curves (AUC) were performed using the R-package pROC [31]. We tested whether AUC of our model-based risk assessment is superior to the baseline alternatives (one-sided test). *p*-values smaller than 5% were considered significant. We also calculated 95% confidence intervals (CI) of AUCs.

## 3. Results

### 3.1. Comparison of Model and Data

**Figure 3:**
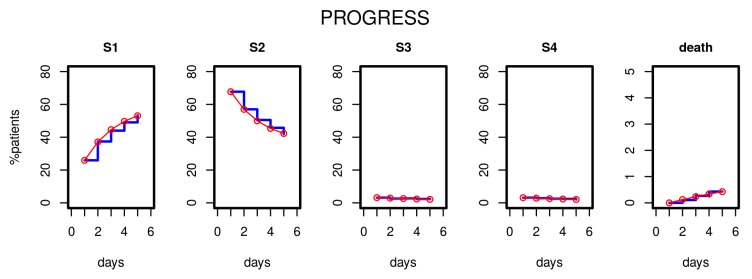
Results of Markov modelling (red curves) in comparison to data of the PROGRESS study (blue curves). Each subfigure shows the proportion of patients in the respective disease states over the first five days. The data fit well with the respective predictions of the Markov model.

After estimating the parameters of our model based on the first five study days, we compared the distribution of disease states over time predicted by the model with those observed in the clinical data. Figure 3 shows the results for the PROGRESS study. Good agreement was observed; i.e., patient time courses were well described by our model. 

**Figure 4:**
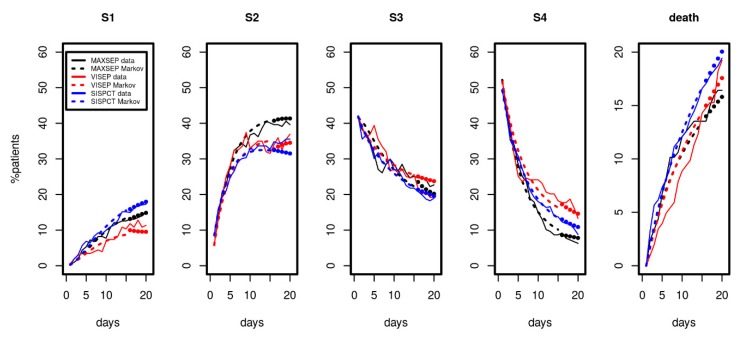
Results of Markov modelling (dashed curves) in comparison to data of the SepNet studies (solid curves) characterized by longer time series. Each panel shows the proportion of patients in the respective disease state over the first 15 days. The filled circles represent model predictions for days not included in model calibration. The data of the three studies fit well with the respective predictions of the Markov model.

Since longer time series were available for the SepNet studies, we considered data from the first 15 observation days for parameter fitting. Results are shown in Figure 4. We again observed a good and uniform agreement of model and data over time, demonstrating that the model can describe the disease course over a longer period. The model even extrapolated well for all disease states and death for days 16 to 20 (see Figure 4, filled circles). 

### 3.2. Transition Probability Matrices

**Figure 5:**
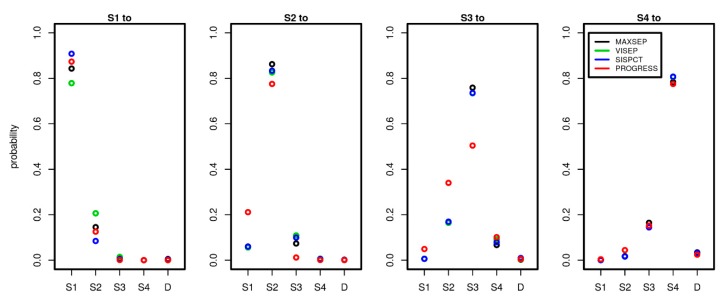
Comparison of transition probabilities between disease states across studies and possible state transitions. To estimate the probabilities, we used 5 observation days for PROGRESS and 15 observation days for all SepNet studies. Each panel shows the daily transition probabilities from one disease state (see panel title) to the other possible disease states shown on the x-axis. Good agreements of transition probabilities between studies were observed.

Since we estimated the transition intensity matrices Q separately for each study, we here compared the results between the studies and dependence on the number of time points used to calibrate the model. To comply with the clinical observations, we considered time periods of one day and compared corresponding transition probabilities between the different disease states. Note that within this period, it is possible that patients experienced multiple transitions in the model, explaining observed transitions S1 to S3 or S1 to S4 etc. Results are shown in Figure 5. 

Remarkably, the estimated transition probabilities showed similar patterns across studies. PROGRESS showed stronger deviations from the SepNet trials, while the SepNet trials showed good agreement of estimated transition probabilities. Moreover, the number of time points used for parameter fitting had no strong impact on the parameter estimates of the SepNet trials (see Appendix A.1, Figure A1). An exception is the VISEP trial, for which stronger heterogeneity of parameter estimates was observed for the transitions S1 to S1 and S1 to S2 in dependence on the number of time points used for model calibration. 

### 3.3. Predicting 28 d Mortality

**Figure 6:**
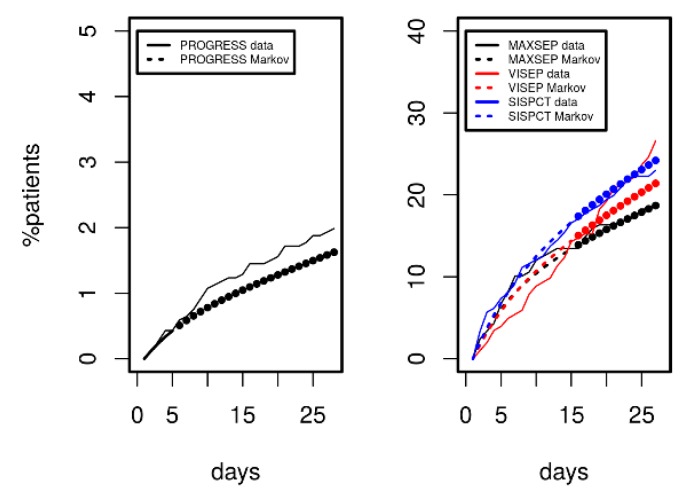
Comparison of mortality predicted by Markov modelling (dashed curves) in comparison to data of PROGRESS and the SepNet studies (solid curves). Agreement between model predictions and data not used for model calibration was excellent (filled circles). VISEP showed slightly inferior prediction.

Since we demonstrated that the model can describe disease courses over a longer time period, we analysed its predictive potential regarding 28 d mortality. Results are shown in Figure 6 for PROGRESS (left panel) and for the three SepNet studies (right panel). Model-predicted mortality was in excellent agreement with observations over a period of 28 days. However, prediction was slightly inferior for VISEP data. 

### 3.4. Distribution of Sojourn Times

**Figure 7:**
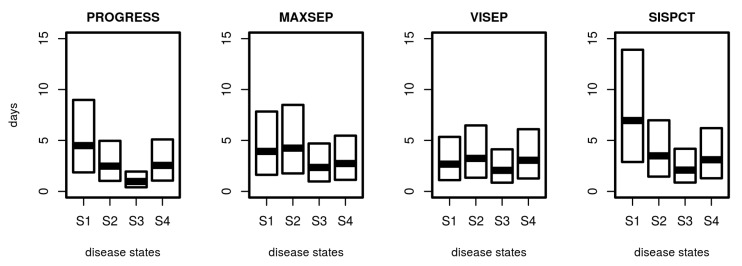
Distribution of sojourn times for disease states. For SepNet studies, we used the transition matrix obtained from fitting the first 15 days. Shown are the median and the 25% and 75% quartile of calculated sojourn times.

Based on the estimated transition intensity matrix *Q*, it is possible to determine the distribution of sojourn times, i.e., the random time a patient stays in a specific disease state. Results are shown in Figure 7. Median sojourn times range between 1 day (PROGRESS, S3) and 7 days (SISPCT, S1). The distribution of sojourn times differed between studies and states. It is shortest for the higher disease states, particularly S3.

### 3.5. Prediction of Death for Patients with Initial Severe CAP

**Figure 8:**
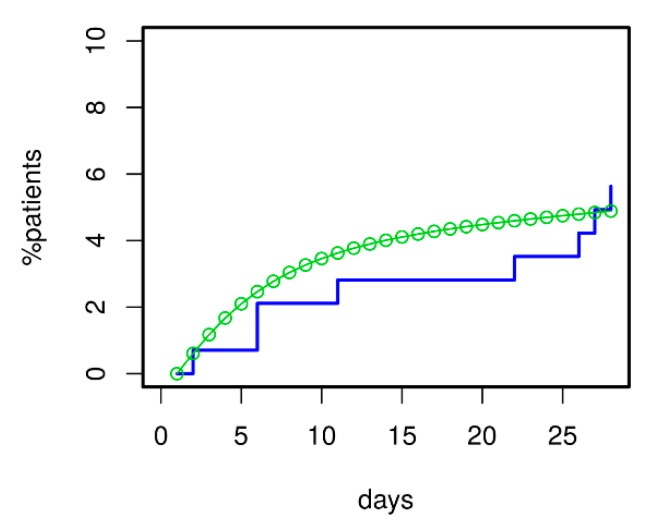
Model validation regarding 28 d mortality was performed using the severely ill CAP patients (ssCAP sub-cohort) of PROGRESS. Predictions and data were in good agreement. Moreover, the model correctly predicted the increased mortality of ssCAP patients compared to PROGRESS patients with mostly lower initial CAP severity used for model calibration (compare with Figure 6).

For validation of the model, we asked how well it could predict 28 d mortality for our ssCAP subcohort of PROGRESS. Data of this cohort were not used for model calibration. Using the transition intensities estimated for PROGRESS, we predicted the time course of mortality for these patients. Predictions were in good agreement with available mortality data (see Figure 8). Moreover, the model correctly predicted that mortality in the validation subset was higher than in the remainder of PROGRESS patients; see Figure 6 for comparison. 

### 3.6. Clinical Utility of the Model

**Figure 9:**
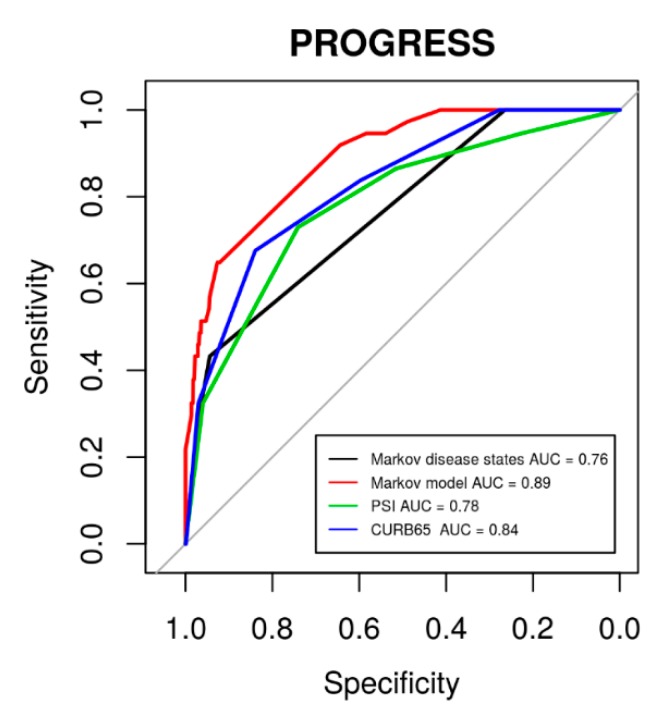
ROC curves for 28 d mortality in the PROGRESS data using established scoring systems PSI (area under curve (AUC) = 0.78, 95% CI: 0.71–0.86) and CURB-65 (AUC = 0.84, 95% CI: 0.76–0.89) in comparison to our initial disease states (AUC = 0.76, 95% CI: 0.70–0.83) and our model-based risk assessment (AUC = 0.89, 95% CI 0.84–0.94).

The major advantage of our modelling is that the individual patient’s risk can be updated, e.g., on a daily scale. To assess this benefit, we compared the corresponding risk evaluation with standard risk evaluation at baseline using CURB-65, PSI, or our defined initial disease states. Results of corresponding ROC analyses regarding 28 d mortality are shown in Figure 9. It revealed that our model-based risk assessment was superior to the baseline risk assessments (baseline disease states: *p* = 5.9 × 10^−4^, PSI: *p* = 6.2 × 10^−3^, CURB-65: *p* = 0.024).

Using the transition probabilities estimated with our model, it is possible to calculate patient’s risk of 28 d mortality in dependence on the current disease state. Results are shown in Table 5, separately for the four studies and in dependence on the current disease state. If, for example, a SISPCT patient is at disease state S1 at baseline, the 28 d mortality risk is 11%. If the patient deteriorates to S3 the next day, the 28 d mortality risk increases to 20%.

## 4. Discussion

In this work, we propose a stochastic mathematical model of the disease course of CAP patients. The model was intended to provide updates of risk assessment for patients at different disease states during their hospital stay regarding disease deterioration and 28 d mortality. Hereby, the model can support monitoring of patients and clinical decision making. The model was developed on the basis of patient time series data taken from four large studies with different initial CAP severity. Model predictions of the evolution of disease states showed good agreement with observed data for the calibration data sets and a validation data set. Parameters estimated on the basis of the different clinical data sets were in good agreement and allowed calculation of clinically interesting characteristics of the disease course, e.g., sojourn times of disease states. 

Initial risk assessment for CAP patients is well established to support decisions on need for hospitalization, e.g., CURB-65 or PSI [5,6,7] or for intensive care treatment, e.g., IDSA/ATS minor criteria [10]. However, although recommended [13], evaluation of changes in risk for deterioration throughout hospitalization is less well established, especially for patients with initially lower risk. We propose to analyze and improve this issue, applying, for the first time, a (stochastic) Markov model of discrete disease states with continuous transition times. Such a model, if successfully applied, would allow risk assessment updates at any time. 

We defined disease states by categories of the SOFA score. Originally, the SOFA score was introduced to describe the sequence of complications in distinct organs [29] and was later shown to be useful for the prediction of outcome in critically ill patients [32]. We showed recently that the SOFA score is also appropriate to characterize disease severity in CAP [24]. To allow reliable estimation of transition rates, it was necessary to summarize SOFA scores into a limited number of disease states with sufficiently high allocation numbers. We here considered five states (four defined by SOFA ranges and death as a possible final state). The first state with SOFA scores less than three was motivated by the observation that almost no deaths are observed in this group of patients. Nevertheless, boundaries and numbers of disease states are arbitrary to some extent. Therefore, we also experimented with four or six states and with different SOFA boundaries of these states. Results of all scenarios were highly similar (not shown). 

In comparison to the model proposed by Rangel-Frausto [23], we refrained from introducing discharge as an independent absorbing state since we observed a number of deaths occurring after hospital discharge and a considerable disease heterogeneity prior to discharge, challenging the assumption that discharged patients are cured completely. Moreover, we do not see an obvious advantage of introducing this state compared to a low-risk disease state. We also refrained from modelling single organ failures separately. Since all patients suffered from CAP, SOFA scores are dominated by the pulmonary subscore. We observed that an isolated model of pulmonary sub-SOFA dynamics can also be established (results not shown). However, this model cannot describe systemic complications during CAP and, therefore, lacks clinical utility to our opinion.

The observed good fit of our data demonstrates that the Markov model assumption is valid for describing disease progression of CAP. This implies that the present disease state determines deterioration risk within the next 24 h, emphasizing the necessity for daily monitoring and re-evaluation of patient’s risk. Our model provides insights into the progression dynamics within patient populations. In particular, estimated transition rates between disease states allow more precise and tailored study designs, for example, regarding selection of patients leading to the required number of deterioration events.

Time-continuous Markov models emerged to be preferable to time-discrete Markov models because they reduce the number of free parameters (transition intensities of non-neighbouring disease states are zero except for death) while still allowing deteriorations of disease states by more than one category per day as observed in our clinical data. Our Markov model is homogeneous in the sense that transition intensities between disease states were assumed constant over time. This assumption is justified in our situation by the observed good agreement of model and data. 

To our knowledge, this is the first application of a stochastic model to describe time courses of CAP. A similar model for sepsis severity was proposed by Rangel-Frausto [23]. Nonrandom dynamical models of sepsis are proposed by Chow et al. [33] and by Zuev et al. [34] but are not applied to clinical time-course data. We previously developed a model of CAP disease course in mice after infection [35], but translation to the human situation proved difficult since the exact time point of infection and data from this early phase are not available.

To calibrate our model, we used time-series data of CAP patients retrieved from four different clinical cohorts. The PROGRESS study is an ongoing observational study enrolling patients hospitalized for CAP. Since the goal of the study was to investigate variability in innate immune response during CAP in immunocompetent patients, study-specific inclusion and exclusion criteria led to a younger and healthier study population with a lower frequency of initially severe CAP and fewer adverse outcomes [25]. To complement PROGRESS data with data from patients with more critical disease courses, we included CAP patients with severe sepsis or septic shock retrieved from three randomized clinical trials of the SepNet study group: MAXSEP, VISEP, and SISPCT [26,27,28]. Although all studies showed a good agreement between model and data, the prediction performance of the model was slightly inferior for the VISEP data. A possible explanation is that here, community-acquired respiratory tract infections were considered; i.e., no working diagnosis of pneumonia was available. Moreover, the study was stopped for safety reasons. 

A limitation of our analysis is that consideration of hospital discharge as independent disease state, probably requiring introduction of hospital readmission, and of patient-specific covariables (e.g., age, sex, comorbidities, or individual biomarkers) was not in scope. Including such covariables into the model by assuming dependence of transition intensities on potentially predictive parameters is generally possible and warrants future investigation. By this concept, risk predictions made by our model could be improved and individualized in the future. 

## 5. Conclusions

We showed that the disease course of CAP patients of different severity follows a time-continuous Markov model; i.e., the deterioration risk of a patient mainly depends on its current state emphasizing the necessity and feasibility of daily updates of risk assessment for CAP patients. The model correctly predicts the rate of mortality over 28 days and therefore paves the way to support clinical decision making and planning of clinical trials. In future work, we plan to refine the model by considering possible dependencies of transition intensities on predictive covariates or biomarkers for improved individualized outcome predictions.

## Figures and Tables

**Table 1:** Study Characteristics. Age and initial Sequential Organ Failure Assessment (SOFA) were reported as medians and interquartile ranges. * Subcohort of PROGRESS patients without study visits.

Study	Age	Sex (m/f)	Initial SOFA	Type of Study	Maximum Observation Time (d)	28d Mortality N (%)	Clinical trial.gov Identifier
PROGRESS	62 (45,74)	1081/782	2 (1,3)	Observational study	5	37 (2.0%)	NCT02782013
ssCAP *	69 (55,76)	104/38	7 (6,8)	Observational study	1	8 (5.6%)	NCT02782013
MAXSEP	67 (57,74)	152/56	10 (8,12)	Randomized trial	22	39 (18.8%)	NCT00534287
VISEP	66 (57,74)	138/65	10 (7.5,12)	Randomized trial	22	54 (26.6%)	NCT00135473
SISPCT	67.5 (56,74)	300/122	9 (7,12)	Randomized Trial	22	97 (23.0%)	NCT00832039

**Table 2:** Disease states defined by the SOFA score.

Disease State	SOFA Score (SC) Range
S1	0 ≤ SC ≤ 2
S2	2 < SC ≤ 5
S3	5 < SC ≤ 9
S4	9 < SC ≤ 24
death	---

**Table 3:** Transitions of disease states observed during the first five days in PROGRESS.

Disease State to From	S1	S2	S3	S4	Death
S1	2545	369	0	0	0
S2	879	3180	49	7	2
S3	1	83	99	18	1
S4	0	5	36	158	5

**Table 4:** Transitions of disease states observed during the first 15 days in the SepNet trials (MAXSEP/VISEP/SISPCT).

Disease State to From	S1	S2	S3	S4	Death
S1	172/109/425	28/28/35	3/3/6	0/0/1	1/0/1
S2	52/47/100	739/664/1308	62/88/155	2/4/5	1/2/3
S3	5/3/6	152/159/323	669/665/1286	60/85/142	2/3/17
S4	0/1/0	8/6/7	120/126/242	548/650/1245	24/24/49

**Table 5:** 28 d mortality risk in dependence on study and disease state.

Study	S1	S2	S3	S4
PROGRESS	0.01	0.01	0.05	0.14
MAXSEP	0.10	0.10	0.13	0.25
VISEP	0.11	0.13	0.17	0.27
SISPCT	0.11	0.15	0.20	0.30

## Appendix A

### A.1. Details of Markov Modelling

We here describe some details of our Markov Modelling. The equation that describes the probability transition matrix (PTM) of such a model is the Chapman Kolmogorov equation (Equation (A1)). In this equation, ***P*** is the transition probability matrix, ***Q*** the transition intensity matrix, and ***t*** is the time. Each entry ***q_ij_*** of the matrix ***Q*** corresponds to the transition rate from disease state ***i*** to disease state ***j***. Since we have five disease states, ***Q*** is a 5x5 matrix. The last row of ***Q*** is zero because death is an absorbing state. Since we consider stepwise deteriorations and improvements of disease state (see Figure 2 in main document), the transition intensity matrix can be further simplified to the structure displayed in Equation (A2). The equation was solved numerically by a method that makes an expansion of the matrix exponential for the transition rates.

We used the R-package *msm* [30] to build the Markov Model. The fitting procedure is based on likelihood maximization for the unknown parameters.

(A1) P(t)=exp(tQ)

(A2) $$Q=\left|\begin{array}{ccccc} q_{11} & q_{12} & q_{13} & q_{14} & q_{15}\\ q_{21} & q_{22} & q_{23} & q_{24} & q_{25}\\ q_{31} & q_{32} & q_{33} & q_{34} & q_{35}\\ q_{41} & q_{42} & q_{43} & q_{44} & q_{45}\\ q_{51} & q_{52} & q_{53} & q_{54} & q_{55} \end{array}\right|=\left|\begin{array}{ccccc} q_{11} & q_{12} & 0 & 0 & q_{15}\\ q_{21} & q_{22} & q_{23} & 0 & q_{25}\\ 0 & q_{32} & q_{33} & q_{34} & q_{35}\\ 0 & 0 & q_{43} & q_{44} & q_{45}\\ 0 & 0 & 0 & 0 & 0 \end{array}\right|$$

## References

[1] IQTIG—Institut für Qualitätssicherung und Transparenz im Gesundheitswesen (2019). Qualitätsreport 2019. https://iqtig.org/downloads/berichte/2018/IQTIG_Qualitaetsreport-2019_2019-09-25.pdf.

[2] Dremsizov T., Clermont G., Kellum J.A., Kalassian K.G., Fine M.J., Angus D.C. (2006). Severe sepsis in community-acquired pneumonia: When does it happen, and do systemic inflammatory response syndrome criteria help predict course?. Chest.

[3] Ewig S., Birkner N., Strauss R., Schaefer E., Pauletzki J., Bischoff H., Schraeder P., Welte T., Hoeffken G. (2009). New perspectives on community-acquired pneumonia in 388 406 patients. Results from a nationwide mandatory performance measurement programme in healthcare quality. Thorax.

[4] Kolditz M., Ewig S., Klapdor B., Schütte H., Winning J., Rupp J., Suttorp N., Welte T., Rohde G. (2015). Community-acquired pneumonia as medical emergency: Predictors of early deterioration. Thorax.

[5] Ewig S., Welte T. (2008). CRB-65 for the assessment of pneumonia severity: Who could ask for more?. Thorax.

[6] Lim W.S. (2003). Defining community acquired pneumonia severity on presentation to hospital: An international derivation and validation study. Thorax.

[7] Fine M.J., Auble T.E., Yealy D.M., Hanusa B.H., Weissfeld L.A., Singer D.E., Coley C.M., Marrie T.J., Kapoor W.N. (1997). A prediction rule to identify low-risk patients with community-acquired pneumonia. N. Engl. J. Med..

[8] Espana P.P., Capelastegui A., Gorordo I., Esteban C., Oribe M., Ortega M., Bilbao A., Quintana J.M. (2006). Development and validation of a clinical prediction rule for severe community-acquired pneumonia. Am. J. Respir. Crit. Care Med..

[9] Charles P.G.P., Wolfe R., Whitby M., Fine M.J., Fuller A.J., Stirling R., Wright A.A., Ramirez J.A., Christiansen K.J., Waterer G.W. (2008). SMART-COP: A tool for predicting the need for intensive respiratory or vasopressor support in community-acquired pneumonia. Clin. Infect. Dis..

[10] Mandell L.A., Wunderink R.G., Anzueto A., Bartlett J.G., Campbell G.D., Dean N.C., Dowell S.F., File T.M., Musher D.M., Niederman M.S. (2007). Infectious Diseases Society of America/American Thoracic Society consensus guidelines on the management of community-acquired pneumonia in adults. Clin. Infect. Dis..

[11] Halm E.A., Fine M.J., Marrie T.J., Coley C.M., Kapoor W.N., Obrosky D.S., Singer D.E. (1998). Time to clinical stability in patients hospitalized with community-acquired pneumonia: Implications for practice guidelines. JAMA.

[12] Akram A.R., Chalmers J.D., Taylor J.K., Rutherford J., Singanayagam A., Hill A.T. (2013). An evaluation of clinical stability criteria to predict hospital course in community-acquired pneumonia. Clin. Microbiol. Infect..

[13] Ewig S., Höffken G., Kern W.V., Rohde G., Flick H., Krause R., Ott S., Bauer T., Dalhoff K., Gatermann S. (2016). Behandlung von erwachsenen Patienten mit ambulant erworbener Pneumonie und Prävention—Update 2016. Pneumologie.

[14] Aliberti S., Amir A., Peyrani P., Mirsaeidi M., Allen M., Moffett B.K., Myers J., Shaib F., Cirino M., Bordon J. (2008). Incidence, etiology, timing, and risk factors for clinical failure in hospitalized patients with community-acquired pneumonia. Chest.

[15] Phua J., Ngerng W.J., Lim T.K. (2010). The impact of a delay in intensive care unit admission for community-acquired pneumonia. Eur. Respir. J..

[16] Peyrani P., Arnold F.W., Bordon J., Furmanek S., Luna C.M., Cavallazzi R., Ramirez J. (2019). Incidence and Mortality of Adults Hospitalized With Community-Acquired Pneumonia According to Clinical Course. Chest.

[17] Chiang C.L. (1976). Making annual indexes of health. Health Serv. Res..

[18] Kay R. (1986). A Markov model for analysing cancer markers and disease states in survival studies. Biometrics.

[19] Klotz J.H., Sharpless L.D. (1994). Estimation for a Markov Heart Transplant Model. J. R. Stat. Soc. Ser. D (Stat.).

[20] Kumar S., Ghildayal N., Ghildayal N. (2017). Markov chain decision model for urinary incontinence procedures. Int. J. Health Care Qual. Assur..

[21] Ieva F., Jackson C.H., Sharples L.D. (2017). Multi-state modelling of repeated hospitalisation and death in patients with heart failure: The use of large administrative databases in clinical epidemiology. Stat. Methods Med. Res..

[22] Mathieu E., Loup P., Dellamonica P., Daures J.P. (2005). Markov modelling of immunological and virological states in HIV-1 infected patients. Biom. J..

[23] Rangel-Frausto M.S., Pittet D., Hwang T., Woolson R.F., Wenzel R.P. (1998). The Dynamics of Disease Progression in Sepsis: Markov Modeling Describing the Natural History and the Likely Impact of Effective Antisepsis Agents. Clin. Infect. Dis..

[24] Ahnert P., Creutz P., Horn K., Schwarzenberger F., Kiehntopf M., Hossain H., Bauer M., Brunkhorst F.M., Reinhart K., Völker U. (2019). Sequential organ failure assessment score is an excellent operationalization of disease severity of adult patients with hospitalized community acquired pneumonia—Results from the prospective observational PROGRESS study. Crit. Care.

[25] Ahnert P., Creutz P., Scholz M., Schütte H., Engel C., Hossain H., Chakraborty T., Bauer M., Kiehntopf M., Völker U. (2016). PROGRESS—Prospective observational study on hospitalized community acquired pneumonia. BMC Pulm. Med..

[26] Brunkhorst F.M., Oppert M., Marx G., Bloos F., Ludewig K., Putensen C., Nierhaus A., Jaschinski U., Meier-Hellmann A., Weyland A. (2012). Effect of empirical treatment with moxifloxacin and meropenem vs meropenem on sepsis-related organ dysfunction in patients with severe sepsis: A randomized trial. JAMA.

[27] Brunkhorst F.M., Engel C., Bloos F., Meier-Hellmann A., Ragaller M., Weiler N., Moerer O., Gruendling M., Oppert M., Grond S. (2008). Intensive insulin therapy and pentastarch resuscitation in severe sepsis. N. Engl. J. Med..

[28] Bloos F., Trips E., Nierhaus A., Briegel J., Heyland D.K., Jaschinski U., Moerer O., Weyland A., Marx G., Gründling M. (2016). Effect of Sodium Selenite Administration and Procalcitonin-Guided Therapy on Mortality in Patients With Severe Sepsis or Septic Shock: A Randomized Clinical Trial. JAMA Intern. Med..

[29] Vincent J.L., Moreno R., Takala J., Willatts S., de Mendonça A., Bruining H., Reinhart C.K., Suter P.M., Thijs L.G. (1996). The SOFA (Sepsis-related Organ Failure Assessment) score to describe organ dysfunction/failure. On behalf of the Working Group on Sepsis-Related Problems of the European Society of Intensive Care Medicine. Intensive Care Med..

[30] Jackson C.H. (2011). Multi-State Models for Panel Data: The msm Package for R. J. Stat. Softw..

[31] Robin X., Turck N., Hainard A., Tiberti N., Lisacek F., Sanchez J.-C., Müller M. (2011). pROC: An open-source package for R and S+ to analyze and compare ROC curves. BMC Bioinform..

[32] Ferreira F.L., Bota D.P., Bross A., Mélot C., Vincent J.L. (2001). Serial evaluation of the SOFA score to predict outcome in critically ill patients. JAMA.

[33] Chow C.C., Clermont G., Kumar R., Lagoa C., Tawadrous Z., Gallo D., Betten B., Bartels J., Constantine G., Fink M.P. (2005). The acute inflammatory response in diverse shock states. Shock.

[34] Zuev S.M., Kingsmore S.F., Gessler D.D.G. (2006). Sepsis progression and outcome: A dynamical model. Theor. Biol. Med. Model..

[35] Schirm S., Ahnert P., Wienhold S., Mueller-Redetzky H., Nouailles-Kursar G., Loeffler M., Witzenrath M., Scholz M. (2016). A Biomathematical Model of Pneumococcal Lung Infection and Antibiotic Treatment in Mice. PLoS ONE.

